# Agreement of Hematologic Measurements Between Jugular and Coccygeal Puncture Sites in Cattle

**DOI:** 10.1111/vcp.70144

**Published:** 2026-08-13

**Authors:** Sophie Clouard, Camille Remuzat, Thomas Chetot

**Affiliations:** ^1^ VetAgro Sup Lyon France; ^2^ Laboratoire Physiopathologie et Génétique du Neurone et du Muscle (PGNM), Institut NeuroMyoGène, INSERM U1315, CNRS UMR 5261 Université Claude Bernard Lyon 1 Lyon France

**Keywords:** blood sampling, cattle, coccygeal vein, concordance, hematology, jugular vein

## Abstract

**Background:**

In bovine medicine, venipuncture is commonly performed at either the jugular or coccygeal vein. Although the coccygeal site is often preferred for practical reasons, sampling sites are inconsistently standardized in scientific studies. It is therefore important to determine whether hematologic values obtained from these two sites can be considered interchangeable.

**Objectives:**

To evaluate the statistical and clinical concordance of hematologic measurands obtained from paired jugular and coccygeal blood samples in cattle.

**Methods:**

Paired jugular and coccygeal blood samples were collected from 48 adult cows and analyzed using an automated hematology analyzer, micro‐hematocrit determination, and blood smear evaluation. Agreement between sites was assessed using Pearson and Lin's correlation coefficients, Passing–Bablok regression, and Bland–Altman analysis, with clinical relevance evaluated against published total allowable error (TEa) thresholds.

**Results:**

Most hematologic measurands showed good agreement between jugular and coccygeal sampling sites. However, platelet counts, packed cell volume (PCV), and certain manual leukocyte differential counts on blood smears, particularly neutrophils and lymphocytes, demonstrated poorer agreement and should not be considered interchangeable.

**Conclusions:**

Jugular and coccygeal puncture sites yield largely comparable hematologic results for most automated measurands. However, platelet indices, packed cell volume, and manual leukocyte differentials on blood smears, particularly neutrophils and lymphocytes, show greater variability between sites and should not be considered interchangeable. In clinical settings, when results are compared with reference intervals or used for longitudinal monitoring, consistent use and documentation of the puncture site are recommended, and reference intervals established using the same sampling site should be applied for these variables.

## 1 Introduction

Blood sampling is an important step for diagnostics in bovine medicine, serving as a critical tool for clinical decision‐making. In field conditions, practical constraints such as animal handling, restraint, and time pressure influence the choice of puncture site. To our knowledge, there are no international guidelines in veterinary medicine regarding the preferred site or technique for cattle venous blood collection. A variety of sampling approaches are routinely employed in practice, depending on the species, context, and operator preference. This lack of standardization underlines the importance of evaluating whether the sampling site can influence hematological results. In routine veterinary practice, coccygeal puncture has gained favor. This is largely attributable to its relative ease, shorter sampling time, and reduced need for head restraint. Despite its practical advantages, we questioned whether coccygeal puncture might influence the analytical reliability of hematologic measurements. To our knowledge, only one study has specifically addressed this question in cattle. Historical data by Parker and Blowey [1] indicated elevated hematocrit and hemoglobin concentration in jugular samples compared to those from the tail vein, but analytical methods at the time lacked the precision of modern hematology analyzers. In other species, including rodents, dogs, and cats, several studies have demonstrated that the site of blood collection may influence certain hematologic parameters, although reported differences are often minimal or clinically negligible [2, 3, 4, 5]. In cattle, existing literature has mainly focused on biochemical analytes rather than hematologic variables when comparing jugular and coccygeal sampling sites [6, 7]. Consequently, the impact of venipuncture site on a broad panel of modern hematologic measurands in cattle remains insufficiently characterized. Given the increasing reliance on automated hematologic assessment and the importance of analytical accuracy in both clinical and research contexts, it is essential to verify whether the site of puncture may introduce statistically or clinically significant bias. This study was designed to compare paired hematologic measurements obtained from the jugular and coccygeal veins of adult cows. We hypothesized that most hematologic measurands would not differ significantly between jugular and coccygeal puncture sites, while analytes known to be sensitive to variations in blood flow, such as platelet counts, might show reduced agreement. The aim of the study was to identify measurands with robust agreement between sites, and conversely, to highlight those for which the sampling location may influence interpretation.

## 2 Materials and Methods

### 2.1 Study Population

The study included 48 adult female cattle, of which 46 were retained for final analysis after exclusion of two animals due to pre‐analytical sample issues (clotting or hemolysis). The cohort comprised 42 presumed healthy animals from two distinct populations: a pedagogical herd maintained at VetAgro Sup (veterinary school) and a local dairy herd, as well as six clinically ill cows hospitalized at the VetAgro Sup bovine clinic. These animals were included to ensure that all sampled animals were represented in the dataset, allowing the study to capture the range of hematologic variation associated with common clinical conditions, while acknowledging that the small number of sick animals is insufficient to draw formal conclusions regarding the effect of a particular disease. All animals represented a range of breeds typical of French dairy and beef herds, including Montbéliarde, Prim'Holstein, Charolaise, and various crossbreeds.

### 2.2 Sampling Procedure

The sequence of blood puncture sites was randomly alternated between animals, with approximately half of the animals sampled first from the jugular site and half from the coccygeal site. Both samples were collected during the same handling session, and the time interval between samplings ranged from 2 to 10 min. Each cow was subjected to two puncture procedures during the same session: one from the jugular vein and another from the coccygeal vein. Jugular venipuncture was executed with the animal secured in a headgate, the neck extended, and the vein visualized by occlusion near the thoracic inlet. An 18G × 1″ needle (Coveto) mounted on a Vacutainer adapter was used (Vacutainer Greiner Bio‐One). For coccygeal sampling, the tail was elevated vertically and punctured at the midline with identical equipment. Blood samples were collected into 4 mL K3‐EDTA tubes (VACUETTE, Greiner Bio‐One) and mixed gently by inversion. They were transported immediately to the laboratory at room temperature. All analyses were completed within 2 h post‐sampling.

### 2.3 Ethical Approval

The study was approved by the VetAgro Sup ethics committee for animal experimentation under authorization number 22103.

### 2.4 Analytical Methods

#### 2.4.1 Automated Hematology

All blood samples were analyzed using a Sysmex XN‐V automated hematology analyzer (Sysmex Corp., Kobe, Japan) with bovine‐specific settings, as recommended by the manufacturer. Although no species‐specific validation studies in cattle have been published for this analyzer, its analytical performance has been evaluated in other veterinary species, including dogs and cats [8, 9], and validation data are available for the closely related Sysmex XT‐2000iV analyzer [10]. In addition, the instrument has been routinely used for bovine blood samples in a diagnostic laboratory for more than 5 years, with regular internal quality control procedures performed. Prior to analysis, tubes containing EDTA‐anticoagulated blood were placed on a hematology tube mixer for approximately 15 min to ensure adequate homogenization, followed by gentle manual inversion. Because bovine‐specific quality control (QC) materials were not available at the time of testing, analyzer performance was verified using the manufacturer's human control material (XN‐Check Level 2, Sysmex). Measured variables included red blood cell count (RBC) by impedance (RBC‐I) or optical (RBC‐O), hemoglobin concentration (HGB), hematocrit (HCT), mean corpuscular volume (MCV), mean corpuscular hemoglobin (MCH), mean corpuscular hemoglobin concentration (MCHC). Total white blood cell (WBC) count and five‐part differential counts (neutrophils, lymphocytes, monocytes, eosinophils, and basophils) were also recorded. Platelet values included impedance (PLT‐I) and optical (PLT‐O) platelet counts. All measurands were recorded from both jugular and coccygeal samples for each animal. The same analyzer and protocol were used throughout the study to minimize analytical variability. Intra‐run coefficients of variation were as follows: RBC‐I and RBC‐O ≤ 1.5%, HGB ≤ 1.0%, HCT ≤ 1.5%, MCV ≤ 1.0%, MCH ≤ 2.0%, MCHC ≤ 2.0%; WBC ≤ 3.0%, NEUT ≤ 8.0%, LYMPH ≤ 8.0%, MONO ≤ 20.0%, EO ≤ 25.0%, BASO ≤ 40.0%; PLT‐I ≤ 4.0%, PLT‐O ≤ 6.0%. These values were used solely to contextualize analytical precision.

#### 2.4.2 Manual Measurements

Packed cell volume (PCV) was determined using the microhematocrit method. EDTA‐anticoagulated blood was drawn into glass capillary tubes, sealed with clay, and centrifuged for 5 min at 12 000 × *g* using a microhematocrit centrifuge.

For morphological evaluation, peripheral blood smears were prepared immediately after sampling, air‐dried, and stained with May‐Grünwald Giemsa stain according to the manufacturer's instructions (DDK S.r.L, Italia). Each smear was evaluated under light microscopy by a single experienced university Veterinary Clinical Pathology instructor, blinded to sampling site, with more than 8 years of professional experience in veterinary hematology. Smears exhibiting inadequate spreading, poor staining quality, or significant artifacts were excluded from further analysis. A 100‐cell manual differential leukocyte count was performed in the monolayer region of the blood smear according to established guidelines [10, 11]. Leukocytes were classified based on bovine lymphocyte morphology, distinguishing small lymphocytes measuring 1–1.5 times the diameter of a red blood cell (RBC), medium lymphocytes 1.5–2.5 times the diameter of an RBC, and large lymphocytes exceeding 2.5 times the diameter of an RBC. Granular lymphocytes, identifiable by the presence of azurophilic granules in their cytoplasm, were included regardless of size.

In addition to leukocyte differentials, each smear was evaluated for platelet aggregation and echinocyte presence using three semiquantitative scoring systems on 10 monolayer fields at ×1000 magnification derived from previously published criteria [12, 13, 14]. Platelet aggregation was assessed using two separate scales: aggregate size score (0, no aggregates; 1, aggregates of 2–5 platelets; 2, aggregates of 6–15 platelets; 3, aggregates of 16–50 platelets; 4, aggregates of > 50 platelets) and aggregate number score (0, no aggregates; 1, < 5 small aggregates; 2, > 5 small aggregates or 1–2 large aggregates; 3, 3–10 large aggregates; 4, > 10 large aggregates). Echinocyte presence was scored from 0 (absent) to 4 (numerous echinocytes observed in multiple fields) (0, none; 1, 1–10 echinocytes; 2, 10–25 echinocytes; 3, 25–50 echinocytes; 4, > 50 echinocytes). These two platelet aggregation complementary scales were used to capture different aspects of platelet clumping: the size score provides an estimate of the biological intensity of aggregation, while the number score assesses the frequency and distribution of aggregates, which is a known predictor of automated counting interference.

### 2.5 Statistical Analysis

Statistical comparisons between jugular and coccygeal sampling sites were conducted using analytical method comparison tools, in accordance with the principles outlined by the American Society for Veterinary Clinical Pathology (ASVCP) [15]. Although these guidelines are intended for the evaluation of analytical methods, they were here adapted to assess the agreement between two sampling sites. The strength of linear correlation between paired measurements was assessed using Pearson correlation coefficients. These coefficients were interpreted as follows: values less than 0.50 were considered weak, values between 0.50 and 0.75 moderate, between 0.75 and 0.90 strong, and values greater than 0.90 indicated very strong correlation. Lin's concordance correlation coefficient (CCC) was calculated to assess the degree to which paired measurements approached the line of identity. CCC values were interpreted from low to high as follows: values of 0.50 or less were regarded as unacceptable; 0.51–0.60 poor; 0.61–0.70 mediocre; 0.71–0.80 satisfactory; 0.81–0.90 good; 0.91–0.95 very good; and values greater than 0.95 indicated excellent concordance.

Passing–Bablok regression analysis was performed to assess concordance between sampling sites. The slope and intercept were evaluated with their 95% confidence intervals; a slope interval containing 1 indicated absence of proportional bias, and an intercept interval containing 0 indicated absence of constant bias. Both conditions met supported interchangeability of methods [16]. Clinical agreement was further assessed using Bland–Altman plots, which provide a graphical representation of the differences between paired measurements relative to their averages, thereby highlighting any systematic bias or variability between methods [17]. In our analysis, jugular venipuncture was considered the reference method, while coccygeal sampling was the comparative method. According to ASVCP guidelines, Bland–Altman analysis is recommended to assess agreement; however, to improve clinical interpretability and relevance, we adapted this approach by applying limits of acceptability based on total allowable error (TEa) values specific to bovine hematology measurands [18]. These limits of acceptability called “limits of Acceptability based on Total Allowable Error” (LoATEa) were calculated as ± (TEa% × mean measurement), thus providing dynamic thresholds that account for variability relative to the magnitude of measurements. Variables were considered clinically interchangeable if at least 95% of paired differences fell within these limits. A mean difference (MeanDiff) confidence interval excluding zero indicates a significant systematic bias exceeding random sampling variability, with the magnitude of MeanDiff estimating the bias extent. In cases where published TEa values were unavailable, Bland–Altman plots were used primarily for qualitative assessment of agreement. Ordinal variables, including platelet aggregate scores and echinocyte scores, were analyzed using the Wilcoxon signed‐rank test for paired samples. This non‐parametric test was chosen because these variables are measured on an ordinal scale rather than a continuous scale, and it appropriately assesses differences between paired measurements from jugular and coccygeal samples.

All statistical analyses were performed using R software (version 4.2.2; R Foundation for Statistical Computing, Vienna, Austria), using base R functions (stats) and, where appropriate, additional packages including DescTools for concordance analyses and ggplot2 for figure preparation. A two‐sided *p*‐value of less than 0.05 was considered statistically significant.

## 3 Results

### 3.1 Study Population and Descriptive Statistics

The study population consisted of 46 adult female cattle, following the exclusion of two animals due to pre‐analytical sample issues (clotting or hemolysis). For Bland–Altman and Passing–Bablok analyses, this number is within the commonly recommended range for evaluating agreement between paired measurements. Previous studies suggest that at least 40 paired samples are sufficient to obtain reliable estimates of limits of agreement and regression parameters [16, 17]. Although formal power calculations are not applicable to these types of agreement analyses, the number of animals included is sufficient to detect major systematic differences and to estimate limits of agreement with reasonable precision. The majority were presumed healthy (*n* = 42) based on a complete clinical examination, including assessment of body condition, vital parameters, and general health status. These animals originated from a pedagogical herd and a local dairy herd. The remaining six cows were clinically ill and hospitalized at the bovine clinic. These animals presented with heterogeneous clinical conditions, including arthritis (*n* = 2), peritonitis (*n* = 1), bronchopneumonia (*n* = 1), and traumatic wounds (*n* = 2). Associated hematologic findings reflected the underlying conditions and included, depending on the individual, mild anemia, erythrocytosis, neutrophilic leukocytosis, monocytosis, lymphopenia, basophilia, or eosinophilia. The number of clinically ill animals does not allow formal evaluation of the effect of disease on venipuncture site agreement. These animals were therefore not included with the intention of assessing disease‐related effects, but to ensure a complete description of the sampled population used for paired concordance analyses. Animals represented a range of breeds commonly found in French dairy and beef herds, including Montbéliarde (*n* = 6), Prim'Holstein (*n* = 32), Charolaise (*n* = 5), and various crossbreeds (*n* = 3). Age ranged from 2 to 5 years (mean ± SD: 3.2 ± 1.6 years). Descriptive statistics for all hematologic measurands are provided in Table 1. For each analyte, mean, minimum, and maximum values are reported separately for jugular and coccygeal puncture. Data distribution was assessed using the Shapiro–Wilk test and visual inspection of histograms and Q–Q plots.

**TABLE 1:** Descriptive statistics of hematologic measurands obtained from jugular and coccygeal venipuncture in adult cattle.

Variables	Unit	Mean jugular	Mean coccygeal	Minimum jugular	Minimum coccygeal	Maximum jugular	Maximum coccygeal	Normality jugular	Normality coccygeal
RBC‐I	×10^12^/L	6.4	6.1	4.6	4.0	7.9	7.6	Yes (0.82)	Yes (0.43)
RBC‐O	×10^12^/L	6.1	5.8	4.5	4.0	7.3	7.2	Yes (0.52)	Yes (0.29)
HGB	g/dL	10.4	10.1	7.8	7.7	12.9	12.3	Yes (0.19)	Yes (0.73)
HCT	%	29.1	28.4	23.7	22	37.5	34.8	Yes (0.12)	Yes (0.50)
MCV	fL	47	46.6	39.3	37.1	54.4	55.2	Yes (0.54)	Yes (0.91)
MCH	pg	16.4	16.7	14.7	14.7	18.2	18.8	Yes (0.26)	Yes (0.32)
MCHC	g/dL	34.9	35.1	32.9	32	36.5	37.2	Yes (0.84)	Yes (0.48)
WBC	×10^9^/L	7.6	7.3	4.3	4.2	11.7	11.6	Yes (0.06)	Yes (0.06)
NEUT	×10^9^/L	2.3	2.3	1.3	1.5	4.0	4.0	Yes (0.25)	Yes (0.09)
LYMPH	×10^9^/L	3.1	3.1	1.3	1.2	5.5	5.4	Yes (0.10)	No (< 0.05)
MONO	×10^9^/L	0.7	0.7	0.4	0.3	1.1	1.2	Yes (0.07)	No (< 0.05)
EO	×10^9^/L	0.7	0.7	0.1	0.1	1.5	1.5	Yes (0.06)	Yes (0.06)
BASO	×10^9^/L	0.1	0.1	0	0.0	0.1	0.1	No (< 0.05)	No (< 0.05)
PLT‐I	×10^9^/L	238	241	151	128	358	388	Yes (0.05)	Yes (0.60)
PLT‐O	×10^9^/L	333	312	217	135	466	460	Yes (0.80)	Yes (0.76)
PCV	%	31	30.5	24	22	49	50	Yes (0.06)	Yes (0.05)
NEUT smear	%	30	31	8	3	65	60	Yes (0.315)	Yes (0.62)
All LYMPH smear	%	55	56	27	29	88	86	Yes (0.90)	Yes (0.34)
Small LYMPH smear	%	12.5	10.6	1	2	32	29	No (< 0.05)	No (< 0.05)
Medium LYMPH smear	%	29.7	27	11	5	57	55	Yes (0.09)	Yes (0.29)
Large LYMPH smear	%	4.2	6.2	1	1	9	14	No (< 0.05)	No (< 0.05)
Granular LYMPH smear	%	1.8	1.9	1	1	4	4	No (< 0.05)	No (< 0.05)
MONO smear	%	3.3	2.8	1	1	8	7	No (< 0.05)	No (< 0.05)
EO smear	%	9.8	9.9	1	1	25	22	No (< 0.05)	No (< 0.05)
BASO smear	%	1.3	1.6	1	1	2	4	No (< 0.05)	No (< 0.05)

*Note:* Normality of each hematologic parameter was assessed using the Shapiro–Wilk test and visual inspection of histograms and Q–Q plots. “Yes” indicates normally distributed (*p* > 0.05), “No” indicates deviation from normality (*p* ≤ 0.05).

### 3.2 Concordance Between Jugular and Coccygeal Sampling Sites for Automated Hematologic Measurands

All concordance results for automated hematologic measurands from the 46 paired samples are summarized in Table 2. Detailed graphical analyses, including Passing–Bablok regression and Bland–Altman plots, are provided as Figures S1 and S2.

**TABLE 2:** Statistical assessment of agreement for hematologic variables between jugular and coccygeal blood samples in cows.

Variables	Pearson correlation coefficient	Lin's concordance correlation coefficient	Passing–Bablok regression slope (95% CI)	Passing–Bablok regression intercept (95% CI)	Percentage of paired results beyond the predefined TEa limits in Bland–Altman analysis
RBC‐I	0.97	0.95	1.03 (0.94–1.14)	−0.37 (−1.04 to 0.19)	4.65
RBC‐O	0.97	0.94	0.99 (0.91–1.12)	−0.17 (−0.90 to 0.33)	4.65
HGB	0.95	0.93	0.94 (0.83–1.05)	0.30 (−0.78 to 1.36)	6.82
HCT	0.96	0.92	0.95 (0.83–1.10)	0.60 (−4.05 to 3.91)	6.98
MCV	0.99	0.99	1.00 (0.97–1.04)	−0.20 (−2.04 to 0.88)	0
MCH	0.99	0.99	1.00 (1.00–1.05)	0.10 (−0.69 to 0.2)	ND
MCHC	0.85	0.79	1.25 (1.00–1.52)	−8.22 (−17.83 to 0.50)	0
WBC	0.99	0.97	0.96 (0.9–1.01)	−0.05 (−0.44 to 0.35)	0
NEUT	0.99	0.99	0.95 (0.90–1.00)	−0.01 (−0.14 to 0.14)	9.30
LYMPH	0.99	0.98	0.94 (0.89–1.00)	0.08 (−0.08 to 0.24)	2.27
MONO	0.99	0.98	0.96 (0.84–1.06)	0.00 (−0.06 to 0.08)	0
EO	0.99	0.99	0.97 (0.91–1.01)	0.00 (−0.02 to 0.03)	0
BASO	0.73	0.70	ND	ND	ND
PLT‐I	0.64	0.62	1.08 (0.85–1.58)	−40.9 (−195.4 to 32.7)	28.3
PLT‐O	0.64	0.61	1.16 (1.00–1.55)	−90.5 (−252.1 to −18.0)	23.9
PCV	0.88	0.86	0.93 (0.71–1.05)	1.00 (−2.00 to 7.43)	18.6
NEUT smear	0.67	0.67	1.07 (0.87–1.63)	−1.61 (−23.0 to 5.75)	26.2
All LYMPH smear	0.71	0.71	1.12 (0.93–1.60)	−7.86 (−30.2 to 3.79)	23.8
Small LYMPH smear	0.68	0.66	0.79 (0.60–1.12)	−0.56 (−4.59 to 1.84)	ND
Medium LYMPH smear	0.66	0.64	1.40 (1.03–2.00)	−7.40 (−21.0 to 1.01)	ND
Large LYMPH smear	0.53	0.50	1.50 (1.00–2.67)	−1.00 (−5.00 to 1.88)	ND
Granular LYMPH smear	ND	ND	ND	ND	ND
MONO smear	ND	ND	ND	ND	ND
EO smear	0.83	0.82	1.00 (0.80–1.32)	0.00 (−1.85 to 1.18)	17.1
BASO smear	ND	ND	ND	ND	ND

Abbreviation: ND, not determined (insufficient paired data due to undetectable values in some samples).

Red blood cell‐related variables such as RBC, HGB, and HCT demonstrated strong linear relationships between jugular and coccygeal sampling sites, with Pearson correlation coefficients exceeding 0.90. Lin's concordance correlation coefficients (CCC) were also high, all above 0.91, indicating very good numerical agreement. For example, the RBC count showed a CCC of 0.95. Passing–Bablok regression analyses supported these findings, with slopes close to 1 and intercepts near zero, suggesting minimal proportional or constant bias between the two sampling sites (Figure S1). White blood cell counts, both total and by differential type, also exhibited strong to excellent concordance between sampling sites. Pearson correlation coefficients were consistently high, with Lin's concordance correlation coefficients (CCC) exceeding 0.95 for most leukocyte populations, indicating excellent numerical agreement. The only exception was basophil counts, which showed lower correlation (Pearson's *r* = 0.725, CCC = 0.703), likely due to the high frequency of zero values (absence of basophils) that precluded Passing–Bablok regression analysis. Platelet variables showed moderate linear correlation with Pearson coefficients ranging from 0.63 to 0.64, but only mediocre to poor agreement according to Lin's concordance correlation coefficients, which ranged from 0.60 to 0.70. Passing–Bablok regression slopes were 1.08 (95% CI: 0.85–1.58) and 1.16 (95% CI: 1.00–1.55), with intercepts between −409 and −90.9, indicating both proportional and constant biases between jugular and coccygeal sampling sites.

Regarding clinical agreement between jugular and coccygeal sampling sites based on total allowable error (LoATEa), red blood cell variables, including RBC and RBC‐O, showed excellent clinical agreement, with only 4.65% of differences exceeding the LoATEa. Hemoglobin (HGB) and hematocrit (HCT) exhibited slightly higher proportions of measurements outside the acceptable limits, at 6.82% and 6.98%, respectively, but remained close to the 5% threshold, suggesting minimal clinical impact. For white blood cell measurands, mean corpuscular hemoglobin concentration (MCHC), total white blood cell count (WBC‐N), monocytes (Mono), and eosinophils (Eo) demonstrated perfect clinical agreement, with 0% of paired differences beyond LoATEa limits. Neutrophils showed slightly lower agreement, with 9.3% of measurements outside acceptable limits, indicating a modest potential for clinical discrepancy. Platelet counts presented the greatest divergence, with 28.3% and 23.9% of measurements outside the LoATEa for platelet count (PLTI) and platelet distribution width (PLTO), respectively, suggesting poor clinical interchangeability between sampling sites for these variables (Figure S2).

### 3.3 Micro‐Hematocrit and Manual Smear

For manual measurements, including PCV and leukocyte differential counts from blood smears, the agreement between jugular and coccygeal sampling sites was more variable. PCV showed a strong correlation, with Pearson and Lin's concordance coefficients of 0.88 and 0.86, respectively. Passing–Bablok regression indicated no proportional bias, but a constant bias was present. Clinically, 18.6% of paired measurements fell outside the LoATEa limits, suggesting limited interchangeability.

For leukocyte differentials, neutrophils demonstrated moderate agreement (Pearson = 0.67, Lin = 0.67), with Passing–Bablok regression revealing a constant bias but no proportional bias. Clinical agreement was lower, with 26.2% of differences exceeding LoATEa limits. Lymphocytes showed similar moderate agreement (Pearson = 0.71, Lin = 0.70) and the same pattern of biases, with 23.8% of measurements outside LoATEa. Eosinophils performed better, showing strong correlation (Pearson = 0.83, Lin = 0.82), no proportional bias, and no constant bias according to Passing–Bablok regression, with only 17.1% of values exceeding clinical acceptability limits. For granular lymphocytes, monocytes, and basophils, correlation analysis could not be performed due to the low number of cells counted, limiting the statistical reliability of these comparisons.

### 3.4 Evaluation of Platelet Aggregates and Echinocyte Scores in Blood Smears

The aggregate size score, representing the average score calculated across five microscopic fields per sample, was slightly higher in jugular samples (median 0.38; 95% CI: 0.19–0.57) compared to coccygeal samples (median 0.30; 95% CI: 0.15–0.45). Similarly, the aggregate number score was somewhat greater in jugular samples (median 0.36; 95% CI: 0.17–0.54) than in coccygeal samples (median 0.30; 95% CI: 0.13–0.36). Echinocyte scores were generally low in both sampling sites, with medians of 0.14 (95% CI: 0.00–0.30) for jugular and 0.11 (95% CI: 0.00–0.23) for coccygeal samples. These scores were assessed on all 41 individuals. Although jugular samples tended to exhibit slightly higher aggregate size and number scores, the overlapping confidence intervals suggest no statistically significant differences exist between sampling sites regarding these artifact‐related variables. The Wilcoxon signed‐rank test revealed no statistically significant differences between jugular and coccygeal samples in aggregate size (*p* = 0.538), aggregate number (*p* = 0.366), or echinocyte scores (*p* = 0.759). This suggests comparable artifact profiles between jugular and coccygeal puncture in bovine blood smears.

## 4 Discussion

This study aimed to compare hematologic measurands obtained from jugular and coccygeal puncture sites in adult cattle, using both automated analyses and manual blood smear evaluations. Overall, our results demonstrate that, for most hematologic variables analyzed by automated methods, jugular and coccygeal sampling yield statistically and clinically comparable results. Several limitations should be considered when interpreting the results of this study. The population was limited to 46 adult female cattle, predominantly Prim'Holstein and Montbéliarde, which restricts generalization to other breeds, sexes, or age groups. Age and lactation stage may influence hematologic parameters, and their potential effects cannot be fully disentangled in this cohort. The study was conducted using a laboratory‐grade hematology analyzer (Sysmex XT‐2000iV), which, although widely used in research and diagnostic laboratories, is not typically available in clinical practice. Therefore, the findings may not fully reflect conditions encountered in field settings. Only jugular and coccygeal puncture sites were evaluated, and intra‐individual variability was not assessed through repeated sampling. Finally, two animals were excluded due to pre‐analytical sample issues, which may have slightly influenced the overall dataset. Despite these limitations, the study provides a comprehensive description of hematologic agreement between jugular and coccygeal puncture in adult cattle.

The results obtained confirm that the site of blood sampling can influence certain hematologic measurands, consistent with observations reported in the literature. Similar to findings in mice and rats, significant differences were noted depending on the sampling site [19, 20]. In dogs and cats, several studies have highlighted minimal or clinically negligible differences between venous sites of varying sizes, such as the jugular, cephalic, and marginal ear veins [2, 3, 4, 5]. In cattle, the literature emphasizes that the coccygeal vein is preferable for certain assays, such as phosphorus, potassium, and phosphate concentrations, due to less interference from surrounding tissues [6, 7]. The study by Parker and Blowey [1], a pioneering work in this field, focused on a limited set of haematologic variables (HCT and HGB) using the analytical methods available at that time and reported significant differences between jugular and coccygeal samples. Our study expands on this groundwork by analyzing a broader range of measurands with modern automated technologies, allowing a more detailed assessment of site‐related variation. Consistent with Parker and Blowey, we observed higher HCT and HGB in jugular samples, and extended these observations to additional variables. Manual blood smear variables, including leukocyte subtyping, showed considerable inter‐site variation, likely reflecting a combination of technical limitations (low cell counts, operator subjectivity, site‐specific artifacts) and genuine biological variation, such as microvascular influences. The lower concordance for basophil counts is mainly due to their low frequency in peripheral blood, limiting statistical reliability.

The most striking lack of agreement in the present study concerned platelet counts. First, platelets are highly sensitive to activation and aggregation during sampling and handling [21]. Jugular venipuncture, which requires more restraint and vessel compression, may provoke platelet activation, leading to artefactual aggregation and falsely altered counts. Second, differences in local hemodynamic shear stress and endothelial surface interaction between the two sites could influence platelet reactivity [22, 23]. Peripheral veins such as the coccygeal may provide a more laminar flow profile, potentially reducing platelet activation compared to jugular sampling. Although differences in platelet aggregation between sites could explain poor concordance, our aggregate scoring showed no significant site‐related differences, and Bland–Altman analysis indicated slightly higher counts in jugular samples, contrary to expectations. Echinocyte scores were low and comparable between sites, suggesting minimal impact of pre‐analytical RBC artifacts. Overall, these results indicate that the observed discrepancies in platelet counts are unlikely due to aggregation or handling artifacts alone and may reflect inherent biological variability or other unmeasured pre‐analytical factors.

A small but consistent increase in cellular counts and indices was observed in jugular samples compared to coccygeal ones (see Bland and Altman figures). This pattern was most pronounced for red blood cells, while white blood cells and platelets showed smaller or less consistent differences. One plausible explanation for these findings is the concept of “plasma skimming”; a characterized hemodynamic phenomenon primarily described at the microcirculatory level, occurring at small vessel bifurcations [24, 25]. It involves the preferential distribution of red blood cells into larger branches, resulting in a higher concentration of red blood cells centrally and a lower concentration in smaller branches. This hypothesis was previously suggested by Parker and Blowey [1]. Although plasma skimming classically concerns microcirculation, here, this concept is applied to larger vessels (jugular and coccygeal veins) to explain the systematic increases in cellular counts observed centrally in jugular samples relative to coccygeal ones. Moreover, the literature suggests that other blood elements, such as other blood cells, may be less affected by this phenomenon due to differences in size and hydrodynamic behaviors, which corresponds well with our observation of smaller differences in these cell types between the two sampling site [26, 27]. Differences in venous return and vascular flow may also contribute. The jugular, a central vein, experiences higher hydrostatic pressure, whereas the peripheral coccygeal vein is exposed to lower perfusion and may be more influenced by local vasomotor tone, temperature, and sympathetic regulation. These factors could subtly affect cellular concentrations. To our knowledge, no studies have directly examined these hemodynamic differences in cattle, and this hypothesis warrants further investigation.

The possibility of inadvertent arterial admixture during coccygeal sampling should be considered. Despite efforts to avoid arterial puncture, the proximity of the coccygeal artery may result in partial arterial contribution, as suggested by the brighter coloration of some tail samples. Arterial blood, with slightly lower hematocrit and hemoglobin, could explain the reduced values observed in coccygeal samples. Similar observations have been reported in rodents, where differences in red cell indices were partly attributed to variable arterial–venous admixture [19, 20].

Six clinically ill cows hospitalized at the VetAgro Sup bovine clinic were included alongside the presumed healthy cohort. Their inclusion was not intended to formally assess the effect of disease on agreement between venipuncture sites, as the number of affected animals was insufficient for such an evaluation. Visual inspection of the paired data showed that these animals were interspersed within the overall dataset and did not consistently exhibit extreme or discordant values compared with healthy individuals. Nevertheless, no conclusions can be drawn regarding the influence of clinical disease on site agreement. Dedicated studies including larger numbers of animals with well‐characterized disease conditions would be required to determine whether pathological states affect hematologic agreement between sampling sites.

From a clinical perspective, all findings have practical implications. For most routine evaluations such as screening for anemia, infection, or systemic inflammation, the coccygeal vein provides results comparable to those from the jugular vein. Given that the sampling site is inconsistently reported or varies across studies establishing bovine reference intervals, our findings suggest that such variability is unlikely to have a meaningful effect on hematologic values [10, 28, 29, 30, 31, 32, 33]. This supports the use of existing reference intervals regardless of whether blood is collected from the jugular or coccygeal vein, which is particularly relevant in field conditions where coccygeal sampling is easier and less stressful. However, for precise platelet counts or detailed leukocyte phenotyping, consistency in the sampling site is recommended to reduce variability. In research settings, standardizing the site is important for reproducibility and for correctly interpreting longitudinal changes, such as disease progression or hematologic response to therapy.

## 5 Conclusions

In adult bovines, blood samples collected from the jugular and coccygeal veins yield hematologic values that are statistically and clinically interchangeable for most variables measured by automated analyzers. However, platelet indices and smear‐based evaluations demonstrate substantial variability and must be interpreted with caution when the sample site is not consistent. Coccygeal puncture remains a reliable and practical option for most clinical applications. These findings reinforce the practical utility of coccygeal puncture in routine veterinary practice, particularly in field conditions where ease of access and minimal animal restraint are important considerations. Standardization of puncture site should be considered in both clinical practice and research contexts to ensure diagnostic precision and comparability.

## Funding

This work was supported by VetAgro Sup.

## Conflicts of Interest

The authors declare no conflicts of interest.

## Supporting information


**Figure S1:** Passing–Bablok regression plots for selected hematologic variables. Passing–Bablok regression plots comparing jugular and coccygeal measurements for selected hematologic variables. Jugular values are plotted on the *x*‐axis and coccygeal values on the *y*‐axis. The red dashed line represents the line of identity. The solid blue line represents the Passing–Bablok regression, with the 95% confidence interval shown as a light‐blue shaded area. Pearson correlation coefficients are reported for descriptive purposes.

**Figure S2:** Bland–Altman plots for selected hematologic variables. Bland–Altman plots showing the agreement between paired jugular and coccygeal measurements for selected hematologic variables. The difference between paired measurements is plotted on the *y*‐axis against their mean on the *x*‐axis. The solid line indicates the mean difference, and dashed lines indicate the 95% limits of agreement. Blue, green, and orange lines represent Allowable Total Error (TEa)—based limits of clinical acceptability according to Nabity et al. Blue lines represent a single constant TEa limit. For Monocytes, blue lines represent within‐reference interval values and green lines represent high values. For Eosinophils, blue lines represent within‐reference interval and high values, whereas orange lines represent low values.

## Data Availability

The data that support the findings of this study are available from the corresponding author upon reasonable request.
