# Supplementary material for: Agreement of Hematologic Measurements Between Jugular and Coccygeal Puncture Sites in Cattle
Source: Vet Clin Pathol. 2026 Aug 13;55(3):295–303. doi: 10.1111/vcp.70144 (PMC13730992; doi:10.1111/vcp.70144)
Supplement: Supplementary file 1 [file VCP-55-295-s001.pdf]

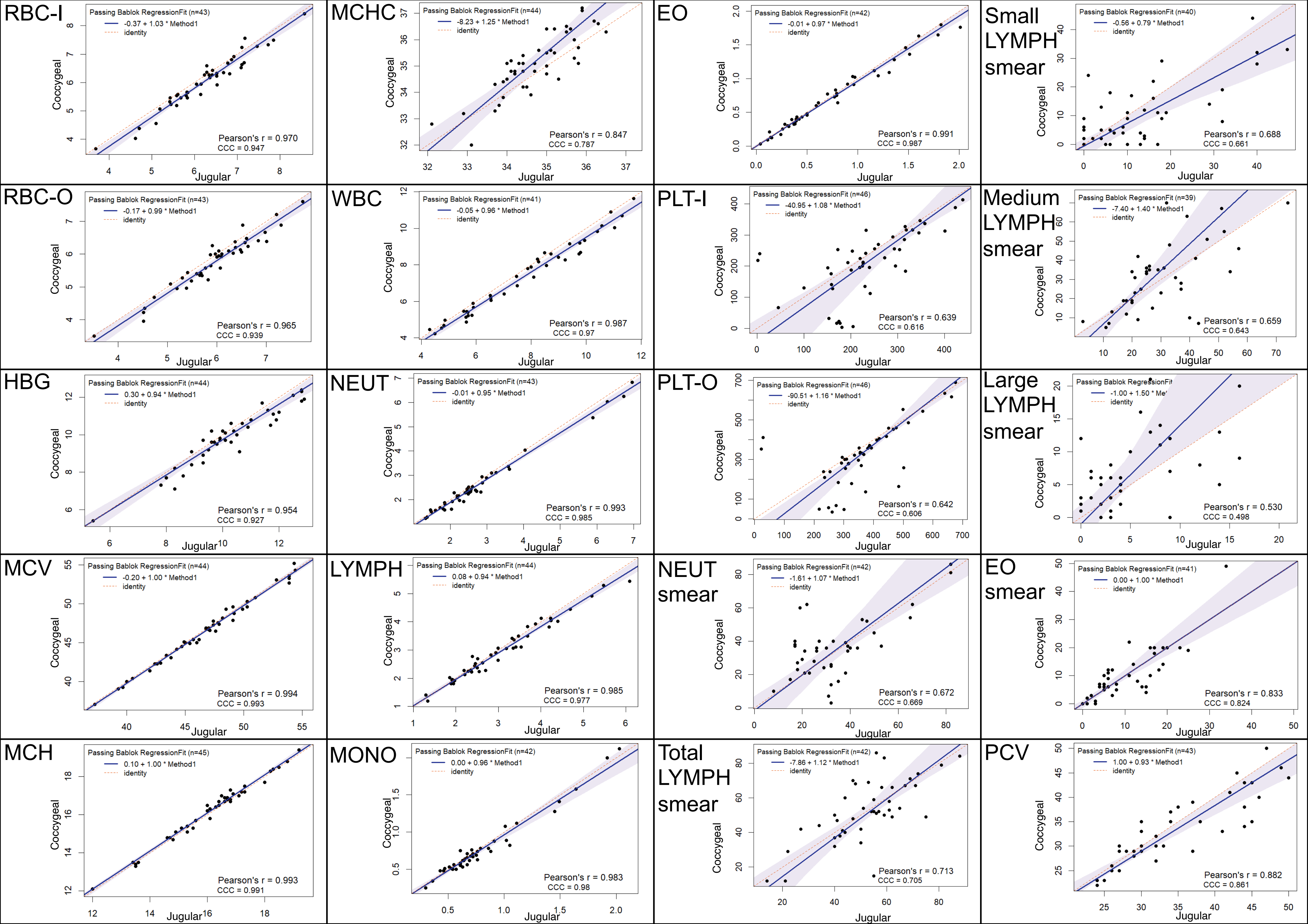
RBC-I
Passing Bablok RegressionFit (n=43)
-0.37 + 1.03 * Method1
identity
Pearson's r = 0.970
CCC = 0.947
Coccygeal
Jugular
MCHC
Passing Bablok RegressionFit (n=44)
-8.23 + 1.25 * Method1
identity
Pearson's r = 0.847
CCC = 0.787
Coccygeal
Jugular
EO
Passing Bablok RegressionFit (n=42)
-0.01 + 0.97 * Method1
identity
Pearson's r = 0.991
CCC = 0.987
Coccygeal
Jugular
Small LYMPH smear
Passing Bablok RegressionFit (n=40)
-0.56 + 0.79 * Method1
identity
Pearson's r = 0.688
CCC = 0.661
Coccygeal
Jugular
RBC-O
Passing Bablok RegressionFit (n=43)
-0.17 + 0.99 * Method1
identity
Pearson's r = 0.965
CCC = 0.939
Coccygeal
Jugular
WBC
Passing Bablok RegressionFit (n=41)
-0.05 + 0.96 * Method1
identity
Pearson's r = 0.987
CCC = 0.97
Coccygeal
Jugular
PLT-I
Passing Bablok RegressionFit (n=46)
-40.95 + 1.08 * Method1
identity
Pearson's r = 0.639
CCC = 0.616
Coccygeal
Jugular
Medium LYMPH smear
Passing Bablok RegressionFit (n=39)
-7.40 + 1.40 * Method1
identity
Pearson's r = 0.659
CCC = 0.643
Coccygeal
Jugular
HBG
Passing Bablok RegressionFit (n=44)
0.30 + 0.94 * Method1
identity
Pearson's r = 0.954
CCC = 0.927
Coccygeal
Jugular
NEUT
Passing Bablok RegressionFit (n=43)
-0.01 + 0.95 * Method1
identity
Pearson's r = 0.993
CCC = 0.985
Coccygeal
Jugular
PLT-O
Passing Bablok RegressionFit (n=46)
-90.51 + 1.16 * Method1
identity
Pearson's r = 0.642
CCC = 0.606
Coccygeal
Jugular
Large LYMPH smear
Passing Bablok RegressionFit
-1.00 + 1.50 * Me
identity
Pearson's r = 0.530
CCC = 0.498
Coccygeal
Jugular
MCV
Passing Bablok RegressionFit (n=44)
-0.20 + 1.00 * Method1
identity
Pearson's r = 0.994
CCC = 0.993
Coccygeal
Jugular
LYMPH
Passing Bablok RegressionFit (n=44)
0.08 + 0.94 * Method1
identity
Pearson's r = 0.985
CCC = 0.977
Coccygeal
Jugular
NEUT smear
Passing Bablok RegressionFit (n=42)
-1.61 + 1.07 * Method1
identity
Pearson's r = 0.672
CCC = 0.669
Coccygeal
Jugular
EO smear
Passing Bablok RegressionFit (n=41)
0.00 + 1.00 * Method1
identity
Pearson's r = 0.833
CCC = 0.824
Coccygeal
Jugular
MCH
Passing Bablok RegressionFit (n=45)
0.10 + 1.00 * Method1
identity
Pearson's r = 0.993
CCC = 0.991
Coccygeal
Jugular
MONO
Passing Bablok RegressionFit (n=42)
0.00 + 0.96 * Method1
identity
Pearson's r = 0.983
CCC = 0.98
Coccygeal
Jugular
Total LYMPH smear
Passing Bablok RegressionFit (n=42)
-7.86 + 1.12 * Method1
identity
Pearson's r = 0.713
CCC = 0.705
Coccygeal
Jugular
PCV
Passing Bablok RegressionFit (n=43)
1.00 + 0.93 * Method1
identity
Pearson's r = 0.882
CCC = 0.861
Coccygeal
Jugular
