# Supplementary material for: Agreement of Hematologic Measurements Between Jugular and Coccygeal Puncture Sites in Cattle
Source: Vet Clin Pathol. 2026 Aug 13;55(3):295–303. doi: 10.1111/vcp.70144 (PMC13730992; doi:10.1111/vcp.70144)
Supplement: Supplementary file 2 [file VCP-55-295-s002.pdf]

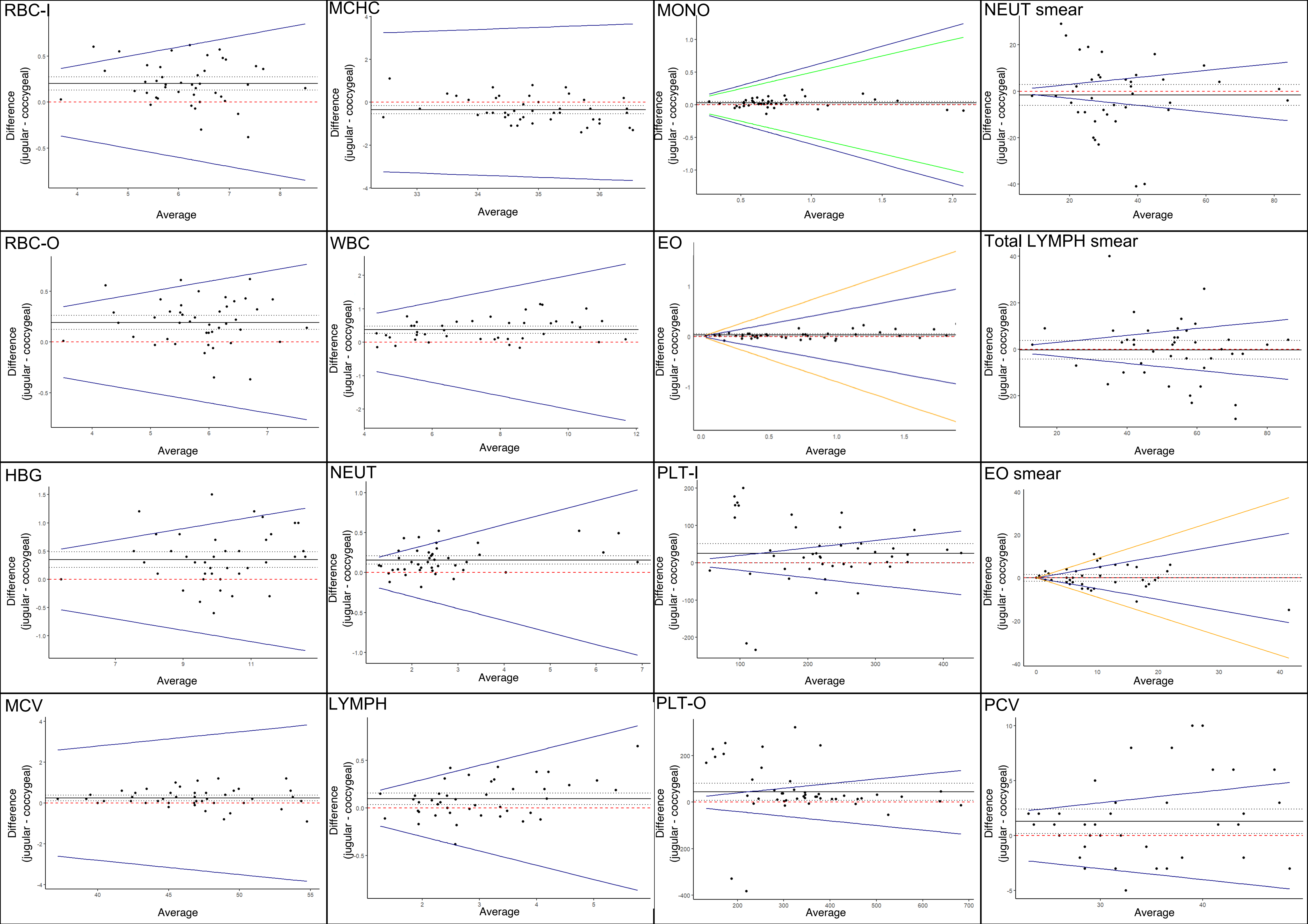
RBC-I
MCHC
MONO
NEUT smear
RBC-O
WBC
EO
Total LYMPH smear
HBG
NEUT
PLT-I
EO smear
MCV
LYMPH
PLT-O
PCV
Difference
(jugular - coccygeal)
Average
